# A TRIZ-driven conceptualisation of finger grip enhancer designs for the elderly

**DOI:** 10.12688/f1000research.51705.1

**Published:** 2021-05-17

**Authors:** Dominic Wen How Tan, Poh Kiat Ng, Ervina Efzan Mhd Noor

**Affiliations:** 1 Faculty of Engineering and Technology, Multimedia University, Jalan Ayer Keroh Lama, Bukit Beruang, Melaka, 75450, Malaysia

**Keywords:** TRIZ, elderly, finger grip, conceptualisation, design

## Abstract

**Background:** Elderly people with severe finger weakness may need assistive health technology interventions. Finger weakness impedes the elderly in executing activities of daily living such as unbuttoning shirts and opening clothes pegs. While studies have related finger weakness with ageing effects, there appears to be no research that uses an algorithmic problem-solving approach such as the theory of inventive problem-solving (TRIZ) to recommend finger grip assistive technologies that resolve the issue of finger weakness among the elderly. Using TRIZ, this study aims to conceptualise finger grip enhancer designs for elderly people.

**Methods:** Several TRIZ tools such as the cause-and-effect chain (CEC) analysis, engineering contradiction, physical contradiction, and substance-field analysis are used to conceptualise solutions that assist elderly people in their day-to-day pinching activities.

**Results:** Based on the segmentation principle, a finger assistant concept powered by a miniature linear actuator is recommended. Specific product development processes are used to further conceptualise the actuation system. The study concluded that the chosen concept should use a DC motor to actuate fingers through tendon cables triggered by a push start button.

**Conclusions: **Finger pinch degradation worsens the quality of life of the elderly. A finger grip enhancer that assists in day-to-day activities may be an effective option for elderly people, not only for their physical but also their mental well-being in society.

## 1. Introduction

The term ‘elderly’ is defined by the United Nations as a person who has reached or exceeded 65 years of age. ^1^ As age progresses, the firing rate of motor units and twitch tension reduces, thus slowing down movements. This occurrence harmfully impacts a person’s muscle health on the whole. ^2^

The increase in the global elderly population leads to challenges in ensuring that elderly people can stay consistently healthy and active. ^3, 4^ Muscle, nerve, and brain degeneration among older people causes finger functions and fine motor skills to deteriorate. ^5, 6^

Researchers have found that elderly people often struggle when loosening or removing the caps of bottles used for storing medicine. ^7, 8^ In connection with this issue, pinching is a task of daily living that many elderly people have trouble with.

A pinch refers to the act of gripping an object with the fingers and thumb with no palmar contact. The usual pinching techniques are the three-jaw-chuck pinch, tip pinch, and lateral pinch. ^9, 10^ While individuals generally use pinching techniques that they are accustomed to using, their exertions of pinch force might be inconsistent across the various techniques. ^11^ Studies have been conducted on various pinch exertion aspects, including a study on the association between grasp stiffness and grasp force. ^12^ The strength of a finger is an underlying component frequently evaluated among elderly stroke survivors. ^13– 15^

It is not uncommon for people to have suffered an injury to the hands or fingers, causing inflammation or pain. Some of these symptoms might have been triggered by wear and tear or overuse injuries from activities of daily living. Aside from injuries, issues in the hands or fingers may also be aggravated by ageing effects. ^16^ This age-related weakness in hand and finger grip strength has been found to significantly reduce elderly people’s quality of life. ^17– 19^

### 1.2. Problem statement

Several studies have explored finger pinch health from different perspectives, including normative data collection of certain populations, ^20– 22^ pinch strength dependent factors, ^23– 25^ and the effect of pinch strength on day-to-day activities. ^26^ Many of these studies are statistically oriented and look into providing basic conjectures on pinching or gripping, along with their effects on people and society. However, there has yet to be a study that uses an algorithmic problem-solving approach, like the theory of inventive problem-solving (TRIZ), to systematically examine pinch function degradation among the elderly and propose innovative solutions for this issue. Hence, using the TRIZ approach, this study aims to conceptualise finger grip enhancer designs that potentially facilitate the day-to-day pinching activities of elderly people.

## 2. Literature review

### 2.1. Age-related changes in the body

The increasing pace of population ageing around the world is concerning as elderly people are prone to having poorer health than young people, and so are more susceptible to mental and physical disorders. ^27, 28^ Elderly people might also experience major health problems with their fingers and hands. For instance, they might experience poor grip and pinch capabilities, which can be a risk factor for carpal tunnel syndrome.

It is likely that one of the contributing factors to hand weakness among the elderly is age-related changes in muscle mass ^29^ and the nervous system. ^30, 31^ Studies have found that by the age of 50, adults experience a reduction in cortical neurons of up to 35 % ^32, 33^ which causes a deterioration of the sensory, motor and even muscle functions. The ageing-induced reduction in strength and skeletal muscle mass is known as sarcopenia. According to Doherty, ^34^ about 30 % of people above the age of 60 experience symptoms of sarcopenia to some extent. This age-related degradation could be caused by a slower pace of life, which could lead to immobilisation and reduced overall muscle health. The number of muscle fibres and the total muscle area decrease with age. ^35^ When this happens, the maximum firing rate of motor units and twitch tension also reduce. ^2, 36^

### 2.2. Factors contributing to reduced pinch abilities

Ageing affects force variation, especially in the application of submaximal force. ^37^ In one particular study, ^38^ elderly people of 65-79 years were found to possess a gripping force 30 % smaller and a peak pinch grip force 26 % smaller than young people of 20-35 years. It was also found that elderly people were less able to sustain a stable submaximal pinch grip force and an accurate pinch position. The study concluded that there is an overall worsening of finger and hand capabilities as people age. ^38^ A positive and significant correlation was found to exist between age and the time required to complete various hand movement subsets. ^39^ Similarly, the decline in hand strength and dexterity was also seen at a smaller scale in the fingers. ^39– 43^

In addition, elderly people with poor eyesight can also experience a weakened finger grip. As an old person’s grip strength slowly reduces, he/she may become dependent on visual feedback. Such a reliance serves to counterbalance the reduced muscular strength whilst engaging with day-to-day tasks. According to Shumway-Cook and Woollacott, ^44^ once people’s eyes are open, their ocular motor system and attention span are triggered to reinforce neuromuscular control and render more sensory input. Vuillerme, Nougier ^45^ found that with vision, people could acclimatise to the destabilisation stemming from muscle fatigue. Vision reduction impacts a person’s proprioception. As proprioception degrades, an individual can slowly fail to perceive precise movements, leading to an increased risk of falling, deteriorating joint disorders, and uncommon joint biomechanics during functional activities. Ageing is connected with impaired proprioception. ^46^

More often than not, elderly people possess reduced hand dexterity and decreased tactile sensitivity. This phenomenon leads to over-gripping in day-to-day tasks. In one study, young and elderly subjects were asked to pinch small objects with varying slipperiness. It was found that the elderly participants exerted double the amount of average force of the young participants. This excessive grip force was a feedback to diminished tactile perception. ^47^

Poor tactile sensitivity reduces the ability of the thumb and fingers to direct force, which is an important factor for securing objects in the hand. ^48– 51^ Young adults were found to have the ability to produce a fingertip force that was nearly perpendicular to the object surface while elderly people produced a force that diverged in the ulnar direction of the vertical plane and proximal direction of the horizontal plane. This outcome adversely affected elderly people’s ability in using hand-held objects as it required the accurate specification of the direction and magnitude of three-dimensional fingertip force vectors.

### 2.3. Methods of improving pinch performance

A common solution for poor finger function is for elderly people to stay regularly active, helping to strengthen muscles and preserve proprioception. Ribeiro and Oliveira ^46^ suggested that keeping oneself physically active on a regular basis could slow down the deterioration of proprioception. Basic long-term finger exercises could lessen fluctuations in the motor output of hand muscles, increase finger dexterity and lower the motor unit discharge rate irregularity. ^52^ Maintaining proper nutrition and performing resistance exercises could also play an important role. ^34^

### 2.4. Basic finger exoskeleton design

An artificial hand is usually required to mimic how the real human hand works. The prosthesis mechanism is analogous to the biological bones of the hand. The synthesis of the finger mechanism is the first step in designing a gripper for the prosthetic arm. The anatomy of the human finger has bones connected by joints (DIP: Distal interphalangeal joint, PIP: proximal interphalangeal joint, MCP: Metacarpophalangeal joint) that are actuated by tendons. ^53^ This anatomy is used as a structure for underactuated systems when coupled with mechanical joints and artificial tendons. There have been studies on human-like prosthetic mechanisms that emphasise the use of underactuated mechanisms. ^54– 56^ An underactuated mechanical system is a system that has a fewer number of control inputs compared to their degrees of freedom (DOFs). These systems have been used in surface vessels, robots, and road vehicles, where the actuators were replaced with passive elastic elements (springs) or limit switches to improve machine adaptability. ^57^

A natural way of grasping an object similar to a human’s grasp can be achieved using an underactuated mechanism. ^57^ This outcome is possible because underactuation allows for adaptive grasping. The geometric configuration of fingers is automatically determined by external constraints related to the shape of the objects. The two main underactuated finger designs utilise linkages and tendon-actuated mechanisms. ^58^ Tendon systems are generally limited to accommodate a small grasping force while linkage mechanisms are suited for a large grasping force.

Although underactuated mechanical systems have been designed for finger prostheses, the concept could be extended to a finger assistive device. The advantage is that it is anthropomorphic, which ensures that the fingers are able to adapt to objects of different shapes. ^58^

### 2.5. Design concept of underactuated devices

In basic underactuated cable mechanisms for prosthetic devices, the fingers function with a thread running through the inner part of the whole finger, which is connected to a servo motor that controls the movement. ^58^ When the finger is flexed, the bottom wire is pulled while the top wire relaxes, and vice versa.

Pons, Rocon ^60^ designed a four-underactuated, multi-fingered dexterous hand which was able to accomplish cylindrical, precision, hook, and lateral grasps. A crossed tendon mechanism was designed to control the fingers. The Federica hand used tendons and pulleys to move its fingers. ^61^ The fingers were connected to tendons actuated by a control pulley mechanism for flexion. Finger extension was achieved with an elastic spring element.

Electronically powered finger prostheses generally use a DC motor-gearhead that requires battery power to drive the motors. ^62^ This external actuated system is usually placed on the forearm, which becomes a challenge in terms of housing all the required components such as the power unit, control unit and actuator. ^63^

## 3. Methods

**Figure 1.**
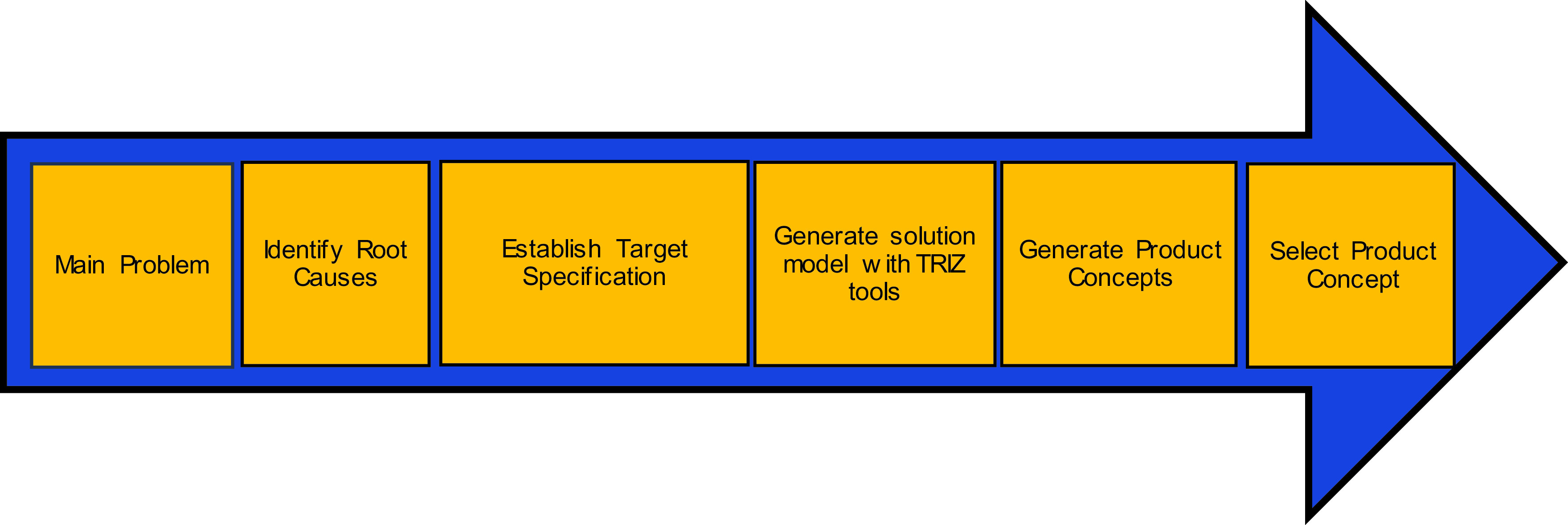
Research methodology flow diagram.

The research methodology flow chart is shown in Figure 1. The main problem is defined by reviewing past literature to establish a cohesive understanding of the current research landscape (see *Underlying data* ^125^ for descriptions of the literature and data sources used). The second step is to identify the root causes of the main problem. In this step, a problem-solving tool known as TRIZ is used. A cause-and-effect chain (CEC) analysis diagram is constructed from key findings of past research to help identify potential causes of the problem and to narrow them down to specific root causes. The method of formulating contradictions is pivotal in TRIZ ^64^ and is applied in the next step. Three methods are used in this research, namely the engineering contradiction, physical contradiction and substance-field analysis. These methods are used in resolving the root causes. After generating the conceptual solutions, the next stage is product development. Selection methods are also applied in various parts of the methodology.

### 3.1 TRIZ methodology

TRIZ is a Russian acronym which stands for the theory of inventive problem-solving. It is an algorithmic problem-solving approach that utilises logic instead of intuition to arouse problem-solving skills inventively. ^65^ The approach involves a methodical process that is predictable, repeatable and reliable. ^66^ It is a systematic problem-solving methodology which originated from patent studies and is suitable for new product innovation.

**Figure 2.**
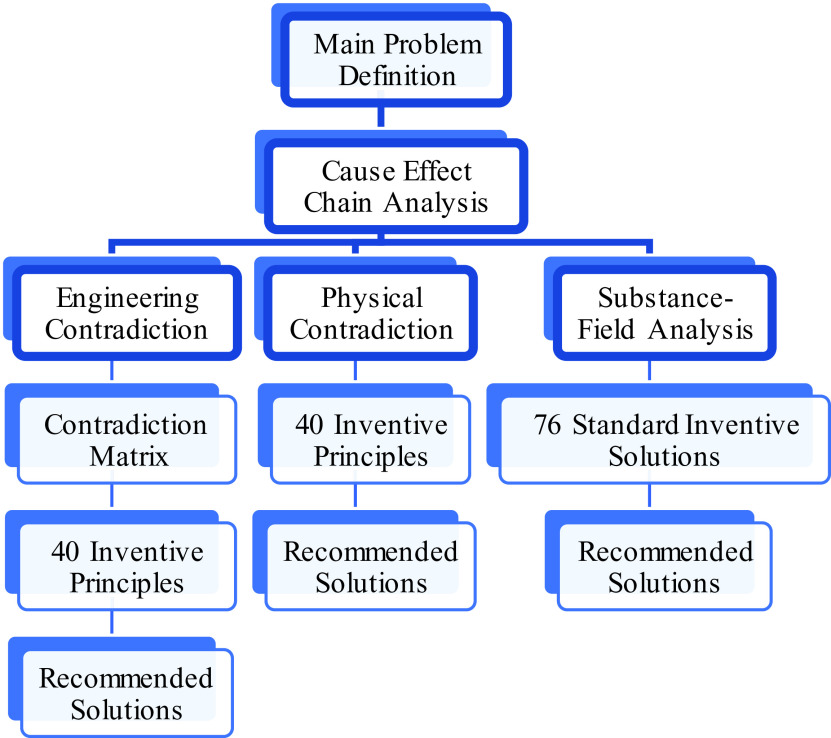
Theory of inventive problem-solving (TRIZ) framework used in this research project.

TRIZ is chosen for this research as it is capable of resolving challenging issues that require problem-solvers to think unconventionally. ^65, 67^ The structure of employing the TRIZ approach for this study is shown in Figure 2. In general, the main problem or initial disadvantage is typically caused by only a few underlying root causes or key disadvantages. The CEC analysis diagram is used to break down the main problem into root causes. In this study, the main problem is identified as “elderly people have degrading finger pinch function”.

*3.1.1. Cause-and-effect chain (CEC) analysis*

**Figure 3.**
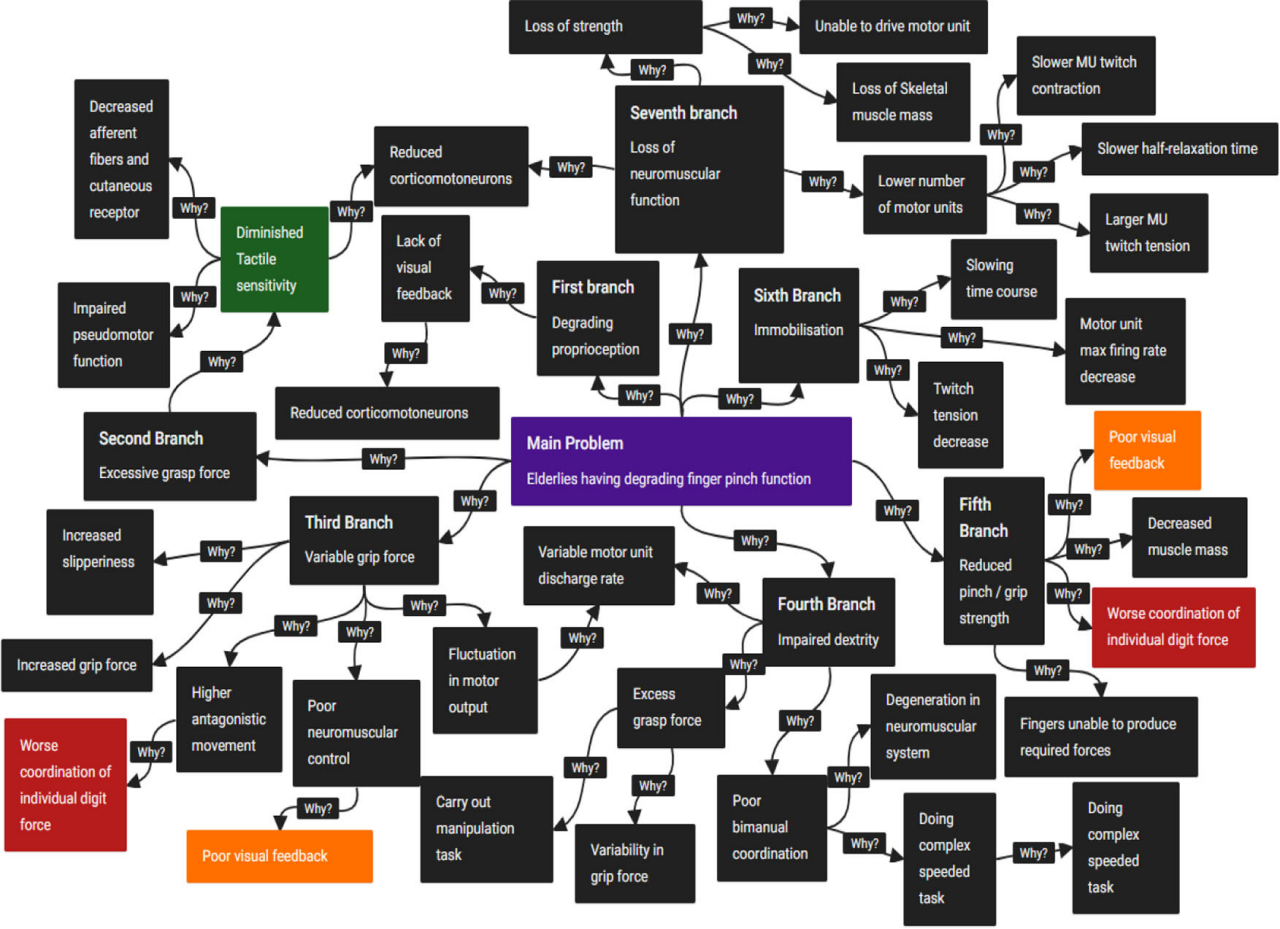
Cause-and-Effect Chain analysis diagram.

The CEC analysis is a systematic method of deriving the cause of a problem. This method helps the researcher focus on the process where an issue has occurred and allows for the practical use of facts to refine the real causes. Starting from the main problem, the question “why” and “why else” is asked until the researcher arrives at a cause that includes a basic theory or law of science, or a cause that has reached a technological limit. The use of literature support is key in the validation of every probable cause. The chain’s end includes a possible root cause to the main problem. Figure 3 shows the CEC diagram constructed for this study.

As shown in the CEC diagram, many different factors contribute to the degrading finger pinching functions of the elderly. Nonetheless, since there are many studies pertaining to the second branch (excessive grasp strength), third branch (variable grip force) and fifth branch (reduced pinch/grip strength) (see Table 1), the exploration for the probable root cause to the problem is refined here for a more focused study.

**Table 1.** Literature citation for cause-and-effect chain root causes.

Root causes	Source examples
Excessive grasp strength	^47, 38, 42, 68^
Variable grip force	^47, 38, 42, 69, 44, 37, 70^
Reduced pinch strength	^38, 42, 71, 72, 73, 74^

The coloured boxes in the CEC diagram are the chosen root causes of the sub-problems. The root causes are basic key disadvantages, recurring across dissimilar branches and convincingly supported by scholarly research. Towards the end of the second branch, the causes point towards fundamental biological factors which would be beyond the scope of this study as far as resolving the main problem is concerned. Therefore, by falling back one level, i.e. to the cause before that branch, diminished tactile sensitivity is selected as one of the root causes. Thus, at the end of the CEC analysis, the following root causes are obtained:

Root cause 1: Poor-visual feedback (yellow box in Figure 3)
Root cause 2: Worsening coordination of individual digit force (red box in Figure 3)
Root cause 3: Diminished tactile sensitivity (green box in Figure 3)

*3.1.2. Engineering contradiction*

After identifying the root causes, various tools in the TRIZ database can be utilised in the next problem-solving step. Inventive principles were defined by Genrich Altshuller ^75^ who analysed 40,000 patents and discovered that all engineering problems can be resolved using a limited set of generalised solutions. An engineering contradiction statement describes an improvement in one characteristic of a system which results in the degradation of another characteristic.

Upon creating the engineering contradictions, proposed recommendations can be filtered by identifying the suitable inventive principles of TRIZ. The inventive principles of TRIZ include a set of 40 generalised recommendations which can potentially resolve an engineering contradiction that is linked with a set of system parameters. ^65^

With reference to the three root causes, three engineering contradictions are constructed and presented in Table 2 together with their respective system parameters and inventive principles. Some of the 39 system parameters of TRIZ ^76^ are linked with the positive and negative aspects in the engineering contradiction. Using the TRIZ matrix of contradictions, these parameters are intersected to obtain the inventive principles.

**Table 2.** Engineering contradictions and inventive principles of root causes.

Subproblem	Engineering contradiction	System parameters	Inventive principle
1. Poor Visual Feedback	**If** visual feedback substitutions are used while pinching,		
	**Then** elderly people will have more information regarding the required applied force,	#27 Reliability	#10 Preliminary action/Prior action **#28 Mechanics substitution/Another sense**
	**But** may not be accustomed to these substitutions without sufficient practice.	#24 Loss of Information	
2. Worsening coordination of individual digit force	**If** extended “fingers” with latching abilities, which can avoid wrongful application of force (more/less), are used,		
	**Then** elderly people will have better strength for activities of daily living,	#10 Force	*Combination #10 & #11* #18 Mechanical vibration #21 Skipping/Hurrying #11 Beforehand cushioning/Prior cushioning *Combination #10 & #22* #14 Spheroidality/Curvature **#15 Dynamisation**
	**But** the safety of elderly people may be at risk as impaired tactile sensitivity between the finger and device can cause stress to fingers.	#11 Pressure/Stress #22 Loss of Energy	
3. Diminished tactile sensitivity	**If** the elderly person pinches with reduced tactile sensitivity,		
	**Then** they will tend to hold their pinch tightly for a longer duration,	#11 Pressure/Stress #15 Duration of Action of Moving Object	*Combination #11 & #10* #36 Phase transitions #35 Parameter changes #21 Rushing through/Skipping *Combination #15 & #10* #19 Periodic Action #2 Taking Out **#16 Partial action**
	**But** they tend to overexert their pinch grip.	#10 Force	

An automated version of this step is available online. The inventive principles can be auto-generated by entering the system parameters based on the worsening and improving characteristics from the engineering contradiction (refer to www.triz40.com/TRIZ_GB.php). Each combination or pair of system parameters yields a different set of inventive principles.

In an earlier study by ^77^ the above-stated methodology of formulating engineering contradictions were similarly used to explore finger grip degradation in the elderly. While the contradiction for the second root cause presented here (worsening coordination of individual digit force) is similar to the one proposed in the earlier study, the contradiction formulations for the first and third root causes are different.

For the first contradiction, the earlier study’s worsening variable contends that digital displays were not able to effectively transfer information to the elderly. However, the present study’s worsening variable argues that elderly people may not be accustomed to visual feedback substitutions without sufficient practice.

The third contradiction in the present study is entirely different from the one discussed in the earlier study because the identified root cause is also entirely different. The earlier study identified the third root cause as the variability in grip force and motor unit discharge rate. However, further literature reviews suggested that this variability in force was predominantly caused by diminished tactile sensitivity.

In view of the foregoing reasons, it was still necessary to reformulate the contradictions from the earlier study in order to uncover different possibilities in the TRIZ solution models.

*3.1.2.1. Engineering contradiction 1 (mechanics substitution/another sense)*

Elderly people face problems with their hearing and vision capabilities. With degrading eyesight, developing an instrument that can replace visual feedback is essential. ^78, 79^ Presently, dynamometers are the most common tools used to evaluate finger strength, due to the simple operations involved and accuracy level. Nevertheless, these tools either use a digital display or a traditional dial that can turn out to be ineffective if the elderly person suffers from low vision. ^71, 74^ This limitation results in decreased stability control and gripping force. Substitutions to visual feedback are essential for constantly rendering information to users especially when their eyesight starts to deteriorate.

The inventive principle known as mechanics substitution/another sense is an appropriate principle to address this contradiction. This principle revolves around the substitution of mechanical factors with sensory (acoustic, smell, taste or optical), electric or magnetic factors. Poor eyesight greatly impacts finger function. Hence, it is reasonable to consider other modes for elderly people to effortlessly process information regarding finger grips. An arm motor network incorporates audio feedback and responds by altering the force applied as well as it does with visual feedback. ^80^ Through practice, auditory feedback can support daily living tasks and can help in avoiding domestic accidents. Researches have suggested the likelihood of auditory feedback as an alternative to visual sensors. ^81, 82^

An alarm- or buzzer-based auditory feedback may be appropriate in monitoring, assisting, and rendering feedback to elderly people. With pressure sensors attached to fingertips, the buzzer can be designed to activate when the required pinch force is reached. It can also be designed to trigger when the user surpasses the demanded force. This system ensures that the elderly person can pinch with the acceptable amount of force, avoiding finger injuries.

*3.1.2.2. Engineering contradiction 2 (dynamisation)*

While extended “fingers” with latching abilities could assist in improved force applications, the interface between the fingers and the device relies on friction to provide the intended force output. With degrading tactile perception, elderly people may face challenges in operating some devices. One study found that indistinct tactile sensory inputs reduced vertical shear force flexibility and increased interdigit shear force coupling in precision grips in order to assure a stable grip control of an object. ^83^ Li, Wei ^84^ found that tactile deficits increased in the inter-digit centre of pressure distribution, causing the force data to stray from the normal distribution, thus impairing finger torque and force control. ^85^

Dynamisation is defined as the act of optimising the characteristic of an object, process, or external environment. Due to the deteriorating coordination of individual digits, elderly people have issues applying the suitable amount of force needed to carry out daily tasks. For instance, they may overexert or apply an inadequate pinch force which could lead to injury. As an analogy, a special tool known as the NuMuv Grip-Aid was developed so that it could be attached to objects like pens or toothbrushes to help people with grasping issues. ^86^ An appropriate tool could be similarly developed to support elderly people in pinching objects more effectively. With the dynamisation principle, the pinched object could be made to interconnect better with the fingers. By developing a kind of latching mechanism, the object could hook, hold, or grasp onto the fingers, thus allowing users to pinch without relying entirely on finger strength.

*3.1.2.3. Engineering contradiction 3 (partial action)*

Elderly people have been reported to possess reduced tactile sensitivity and hand dexterity. ^87, 88^ This limitation could lead to over-gripping issues during activities of daily living. ^89^ Shim, Lay ^69^ suggested that grip force may be energetically suboptimal but could still benefit the elderly. A larger grip force would stop objects from slipping off the hand even when the grip force fluctuates. Such an application may be useful for less steady and frail elderly people. ^90, 91^ However, this high grip force could be a strategic response to tactile sensitivity impairment which also contributes to impaired dexterity.

A high force produces a stronger pinch grip yet causes more stress on the finger muscles and could lead to long term injuries. The partial action principle refers to the use of more or less of the originally desired action, effort, or field when the exact output is difficult to achieve. ^65^ This strategy is appropriate for helping elderly people avoid overexerting their fingers in a pinch application. The strategy can be accomplished by pinching with less of the initially required force instead of overexerting the force in one shot when the exact intended force level is difficult to achieve. Moreover, researchers discovered that elderly people use a probing pinch strategy, which causes a large force fluctuation. ^91^ Improving friction would indeed allow people to grip more securely, thus allowing for improved pinch force control. An unconventional method of improving pinch force control would include exploring the use of dampers which absorb the added force without compromising the firmness and strength of the pinch.

*3.1.3. Physical contradiction*

A physical contradiction comprises two opposing conditions in a single object. ^92^ These conditions cause a conflicting requirement in the object’s functionality. Compared to an engineering contradiction, a physical contradiction is resolved through separation principles of the contradictory requirements in time, space, or physical state. ^93^ For this study, three physical contradictions are formulated.

Physical contradiction 1:

Elderly people need visual feedback substitutions to be informed about pinch force performance, AND elderly people do not need visual feedback substitutions as injuries may occur from unaccustomed use.

Physical contradiction 2:

Elderly people need to pinch with higher pinch force using a pinching device for better pinching results, AND elderly people need to pinch with reduced pinch force without a pinching device to not injure the fingers with external mechanisms.

Physical contradiction 3:

Elderly people need to pinch with reduced tactile sensitivity in order to hold their pinch tightly for a longer duration, AND elderly people need to pinch with increased tactile sensitivity in order to not overexert their pinch grip.

The approach used to solve all three physical contradictions is the separation-in-space strategy. The strategy is suitable for the first contradiction because there is an operating space to have visual feedback substitutions and an operating space to reduce the risk of the unaccustomed use of these substitutions. In the second contradiction, there is an operating space to have higher pinch force using pinching devices and an operating space to avoid injuring fingers from external mechanisms. In the third contradiction, an operating space exists in pinching firmly and an operating space exists in avoiding overexertion. According to the separation-in-space approach, the inventive principles prescribed to resolve the physical contradictions include:

**Table:** 

#1 Segmentation	#13 The other way around	#4 Asymmetry
#2 Taking out	#14 Curvature	#24 Intermediary
#3 Local quality	#7 Nested doll	#26 Copying
#17 Another dimension	#30 Flexible shells/Thin films	

The taking out principle is selected to resolve physical contradiction 1, while the segmentation principle is selected to resolve physical contradiction 2. The other way around principle is selected to resolve physical contradiction 3. Further deliberation is carried out on these principles for possible recommended solutions.

*3.1.3.1. Physical contradiction 1 (taking out)*

According to Oscari, Secoli, ^80^ it was found that arm motor networks incorporate auditory feedback in achieving desired trajectories. This finding implied that auditory feedback is a suitable sensory substitution in motor training. The taking-out principle is used to separate a particular portion from the main object or isolate a critical part/property from the rest of the object.

With our bodies often relying on visual feedback while performing day-to-day activities, practice would be required for any form of visual substitution. From an experiment by Portnoy, Halaby, ^81^ blindfolded participants who received auditory feedback showed that the water-spilling error (the output variable measured) between the first and last trial was significantly reduced. Based on the taking-out principle, a solution could be proposed to remove the time required for a person to be familiar with any form of visual feedback substitution. Exploring different combinations of sensors could help realise this idea by combining various levels of feedback such as a buzzer (auditory), LED lamp (visual) or vibrating device (kinaesthetic).

*3.1.3.2. Physical contradiction 2 (segmentation)*

Apart from finger strength, finger individuation is a significant component of hand function. Finger individuation is the ability to subtly operate the fingers, something which is often a struggle among stroke patients and elderly people. ^94^ Independent finger movements were found to be restricted as the motor cortex or corticospinal tract deteriorates with age. ^95, 96^ This restriction is predominantly noticeable for the little, middle and ring finger as these fingers were found to be considerably impaired. ^95^

When gripping small objects, one must have the individuation ability to position fingers in the correct orientation and exert enough force to grip the object. ^97^ Understanding the differences in how each finger changes with age could influence the process of developing a finger grip enhancing device. Segmentation is used to divide an object into independent parts. Rather than researching pinch as a general finger action, it would be more suitable (as suggested by past researches ^98, 99^) to refine the action into minor elements of individual fingers. Using this principle in the development of a pinch assistant, more focus should be allotted to weaker fingers like the little, middle and ring fingers to make up for the seriously impaired individuation.

*3.1.3.3. Physical contradiction 3 (the other way around)*

In finger prehension tasks, elderly people were found to produce a higher opposing moment which acts against the direction of total moment. ^69^ Similar to the produced excess force, this action can be viewed as an inefficient gripping method, though it increases stability. Opposing moments increase the apparent stiffness of the hand and can be a passive resistance to variations in torque. In other studies, researchers found that the central nervous system was unable to organise finger synergies such that finger forces cancelled each other out. ^100, 101^ During prehensile tasks, fingers produce a nonzero force prior to the task which acts against the external torque. ^69^ These phenomena occur instinctively in elderly people to increase the safety margins as they use their fingers.

The other way around principle is used to invert actions or reverse processes for problem-solving. Using this principle, it is beneficial to view opposing moments from another perspective. Instead of trying to eliminate these natural, age-related responses of the fingers, factoring these responses into future studies or medical devices helps people age gracefully as their bodies intend.

A device utilising the other way around principle should pay attention to the preloading force of the elderly person’s fingers. Instead of designing something that restricts initial force application entirely, the device should allow fingers to apply a preloading force first before assisting it with mechanisms. This application would allow elderly people to remain active as a countermeasure for expected loss of strength and endurance. A study among elderly people over the age of 90 found that with resistance training, functional mobility improved, while strength increased by up to 175 %, ^102^ suggesting the possibility of reversal of age-related effects on skeletal muscles.

*3.1.4. Substance-field modelling*

The substance-field (Su-Field) analysis is another TRIZ problem-solving tool. It is used to analyse and improve the efficacy of technical systems. The Su-Field mainly consists of a few main fields including the mechanical, acoustic, thermal, chemical, electric, and magnetic fields. A field makes up the most basic model of an interaction. Any interaction can be formulated as the interaction between two substances. ^66^ The interaction between the fingers and a pinched object can be modelled using the Su-Field analysis, with the main problem being that elderly people have degrading finger pinch function. The type of model identified determines the class of standard inventive solutions (from the 76 TRIZ standard inventive solutions) to be used for the modelled problem. ^65^

**Figure 4.**
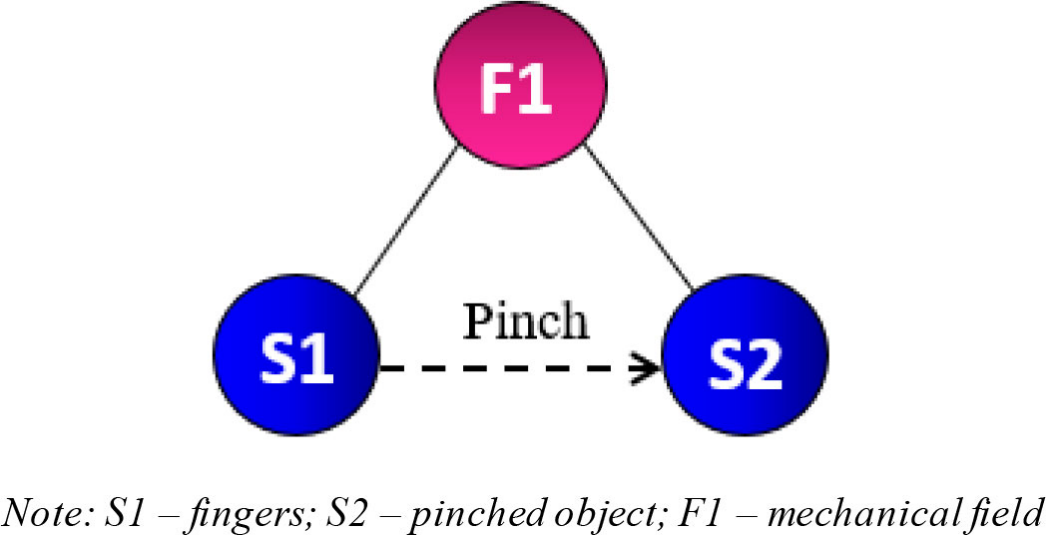
Ineffective Su-Field of finger pinch.

Figure 4 presents the Su-Field model of this study. S1 refers to the first substance, which in this case represents fingers. S2 represents the pinched object. F1 is the mechanical field, which includes the interaction between fingers and objects. This model is known as an ineffective Su-Field model, which is normally represented by a dotted arrow from S1 to S2. The model implies that the fingers are ineffectively pinching the object. From the list of 76 Standard Inventive Solutions, class 2 and 3 are used as a reference to resolve the problem. These classes include various methods of increasing Su-Field effectiveness. Sub-classes 2.1.1 and 2.1.2 are finally chosen as the solution models because the researchers were able to visually conceptualise practical solutions from these two sub-classes.

*3.1.4.1. Chain Su-Field model (2.1.1) - converting single model to chained model*

**Figure 5.**
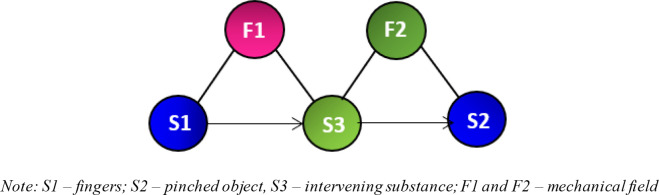
Chain Su-Field model with additional substance and field.

Figure 5 shows a chain Su-Field model, which is a modified version of the previous ineffective Su-Field model ( Figure 4). Another field (F2) and substance (S3) have been added. S3 is added to separate substances S1 and S2. The additional substance should potentially increase the effectiveness of the fingers while pinching. In this scenario, S3 could include the addition of friction between the fingers and pinched object. This proposition, which can take the form of rubber pads or gloves, improves tactile sensibility, leading to a plausible increase in force accuracy and reduced overexertion. F2 represents a mechanical field.

As people age, the tactile sensibility of hands becomes impaired, ^38, 42, 47^ worsening their ability to pinch accurately, moderately and securely. Adding a new substance between the finger and the object can potentially eliminate this effect.

When the finger pinches an object, it slips against the object surface when the direction of the finger force is such that the ratio of shear to normal force becomes larger than the coefficient of friction between the finger and the object. ^103^ Fingers generate forces within a range that is dependent on the coefficient of friction. A low-friction surface would require finger forces to be applied in a direction close to perpendicular to the object surface which would be harder to maintain, resulting in slippage. ^104^

Rubber gloves are popular tools used for increasing friction between the fingers for any hand-holding application. This idea can be incorporated into devices for activities of daily living. A study in 2015 discovered a progressive increase in grip-to-load force during the precision pinch of low-mass objects (less than 30 g). ^105^ The same study discussed the possibility of how a decreased level of tactile feedback played a role in inefficient low-mass grip force control. By considering these findings on tactile sensitivity effects on pinching, a tool can be introduced for the elderly to increase finger friction and reduce the ineffectiveness caused by pinching slippery objects.

*3.1.4.2. Double Su-Field model (2.1.2) - a second field applied to S2*

**Figure 6.**
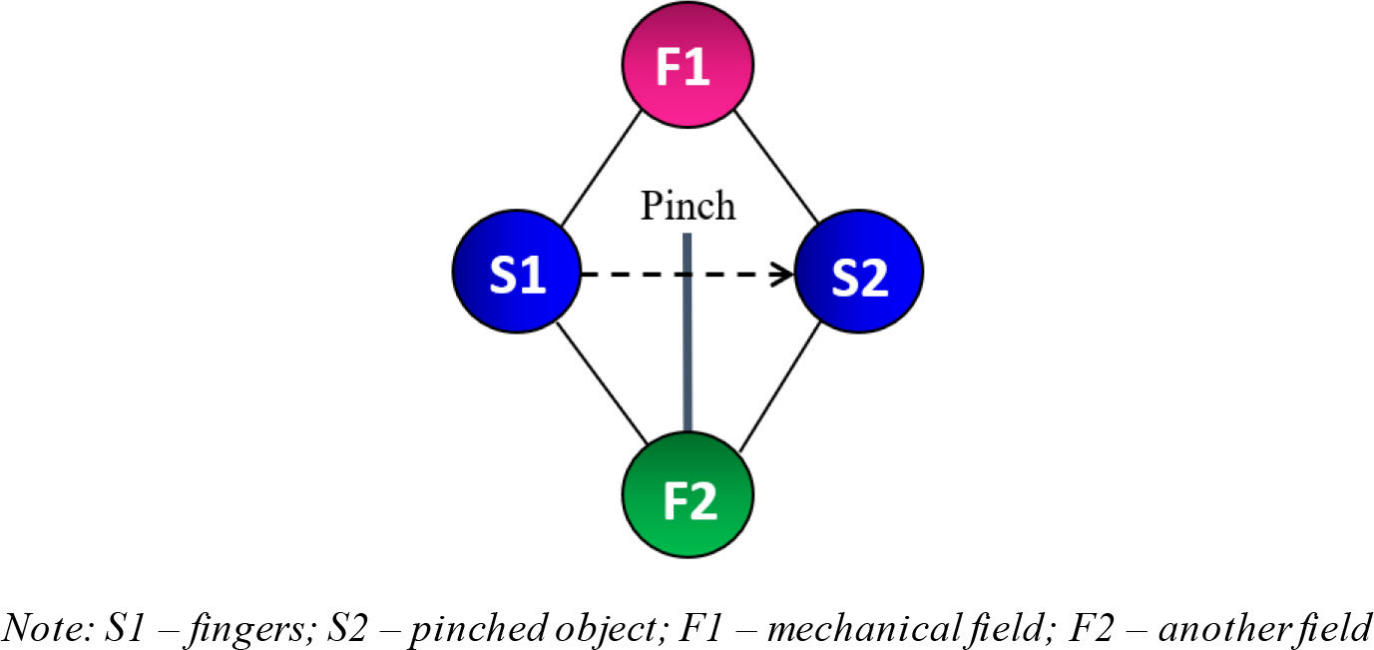
Adding another field for better control of the system.

Figure 6 is a double Su-Field model, which is a modified version of the original ineffective Su-Field model ( Figure 4). Another field (F2) has been added to allow better control of the system. The mechanical field F1 produced by the fingers pinching the object is ineffective. These effects can be improved by adding F2. Some of the possible fields include the optic or acoustic fields. This solution model does not separate S1 and S2 and ensures that the fingers remain in contact with the pinched object. As people age, their eyesight begins to deteriorate. Poor eyesight has a strong correlation with poor finger pinching. ^44, 46, 71^ The incorporation of LED sensors on a device that aids pinching should provide a clear signal to indicate to the user that sufficient force is detected for the pinching action.

It is also important to consider other mediums in which information regarding finger gripping can be easily conveyed to elderly people. Auditory feedback is one of the possible substitutes to visual sensors. ^81, 82^

## 4. Results and discussion

### 4.1. Concept generation

The solution models generated from all three approaches (engineering contradiction, physical contradiction and substance-field analysis) were compiled and used to initiate the concept design process. The following list summarises the generated principles and sub-classes used as solution models.

1. Engineering contradiction:
  a. Mechanics substitution/another sense
  b. Dynamisation
  c. Partial action
2. Physical contradiction:
  a. Taking out
  b. Segmentation
  c. The other way around
3. Substance-field analysis:
  a. Chained Su-Field for independent control of substance
  b. Improving ineffectiveness with F2 without changing elements of system

**Figure 7.**
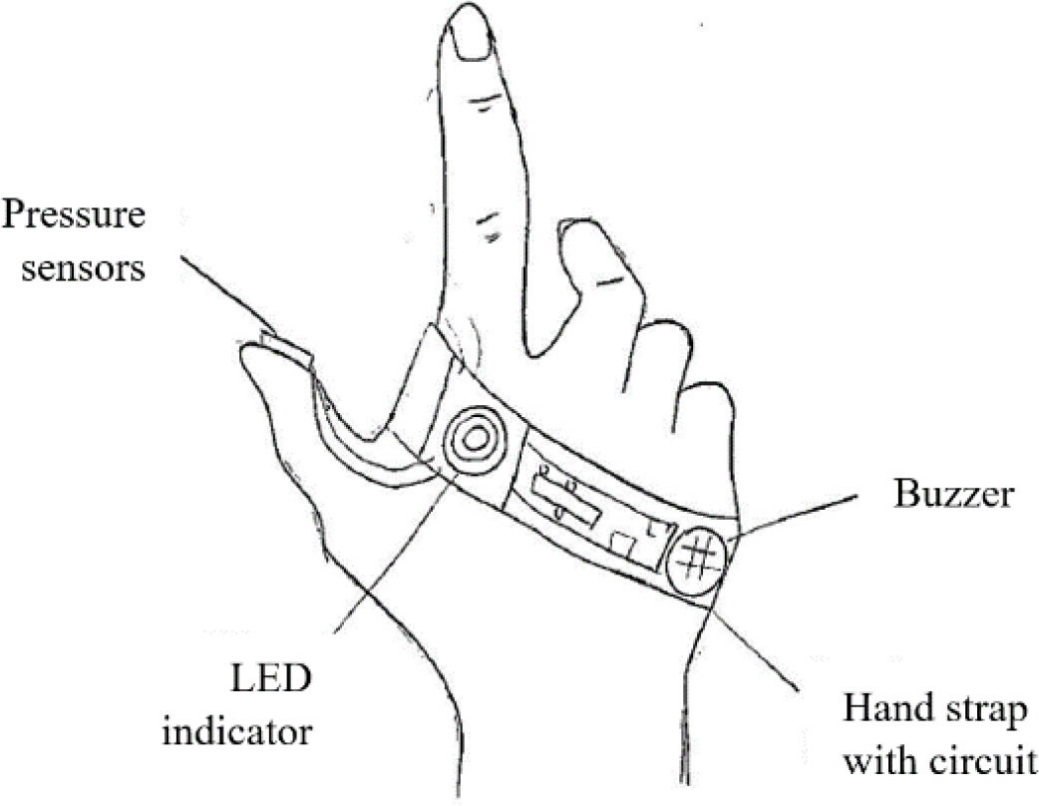
Mechanics substitution/another sense and taking out concept design.

The concept design for the mechanics substitution, another sense, and taking-out principles were combined as shown in Figure 7. The combination included different feedback sensors that notify the user when a sufficient force has been applied while pinching objects.

**Figure 8.**
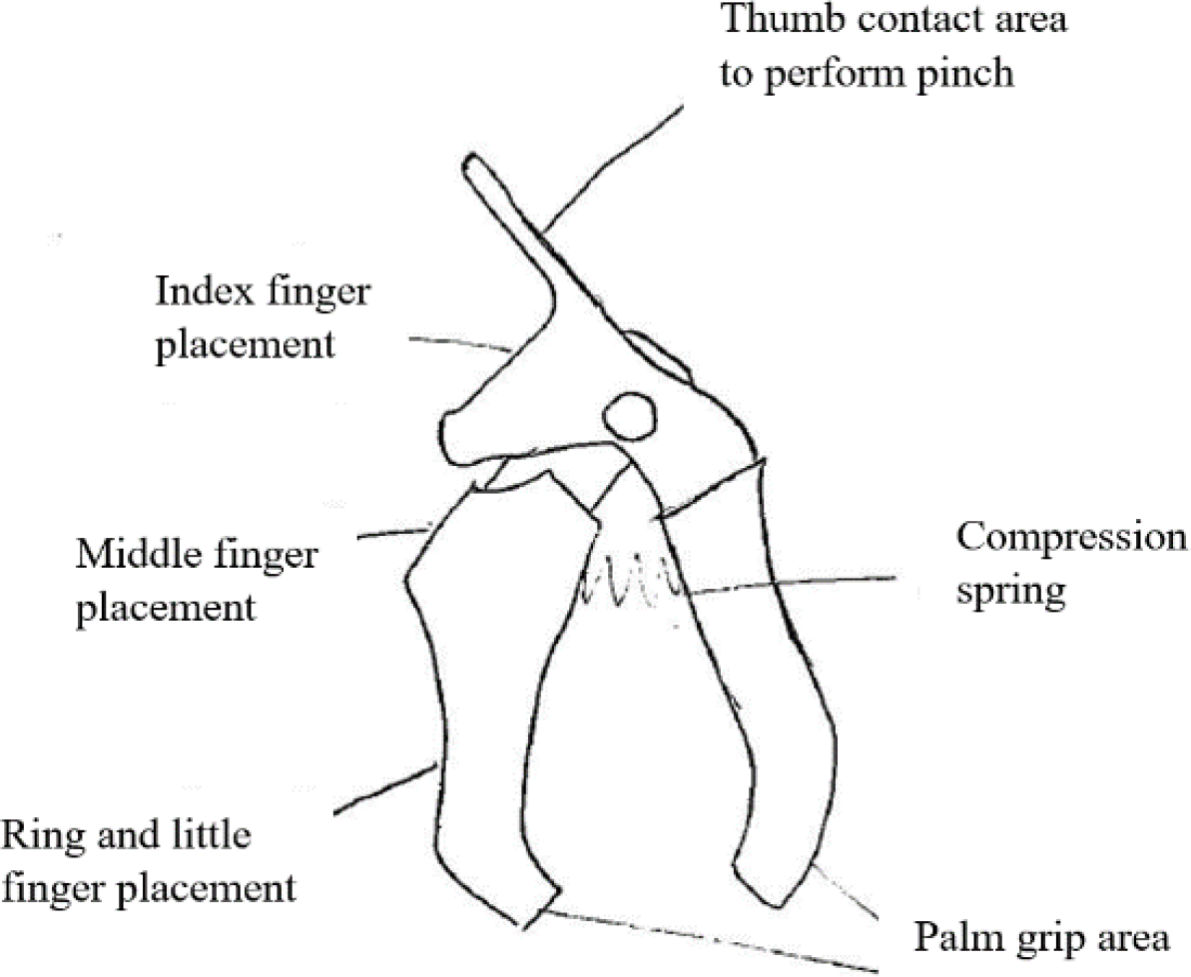
Dynamisation concept design.

**Figure 9.**
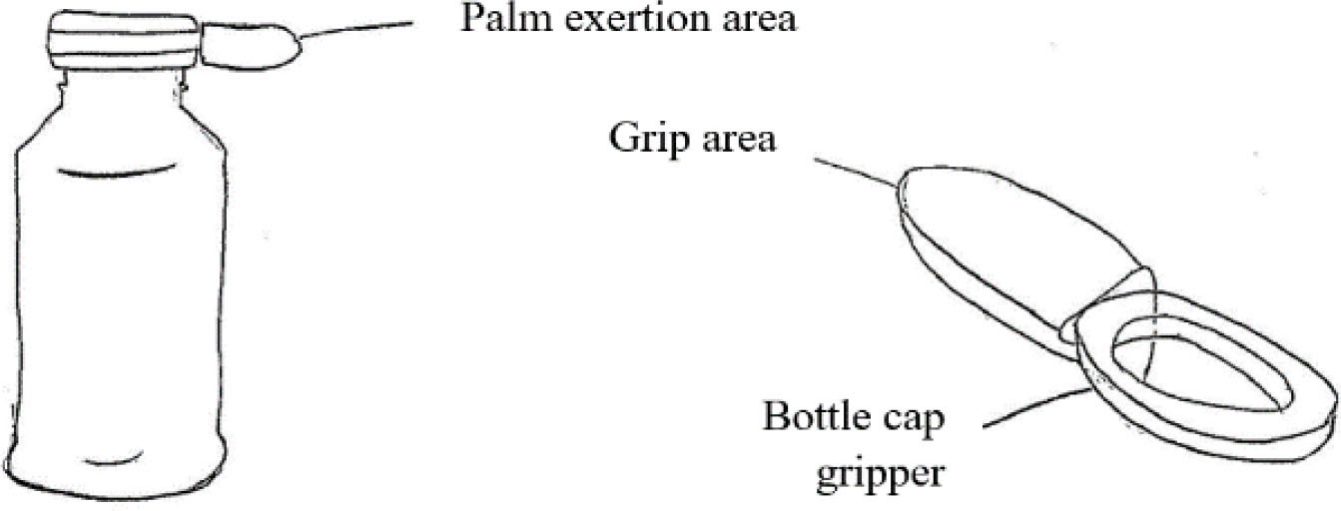
Partial action concept design.

Figure 8 shows a concept based on the dynamisation principle. In this concept, palmar grip, which is normally inactive in the pinch action, would provide grip strength to the fingers during pinching. Figure 9 shows a device specifically used to open bottle caps. For the elderly people found to be using a probing pinch approach, the large contact area of this device would allow them to use the palm for more force exertion and an easier twisting action due to the increase in the moment of the force.

**Figure 10.**
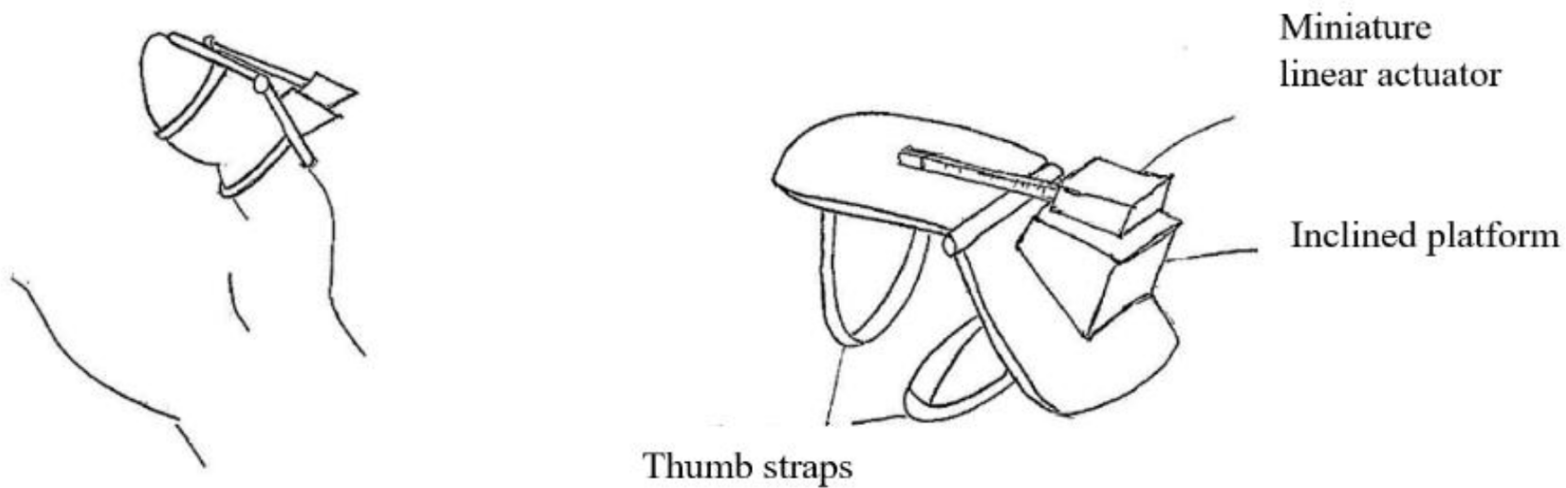
Segmentation concept design.

**Figure 11.**
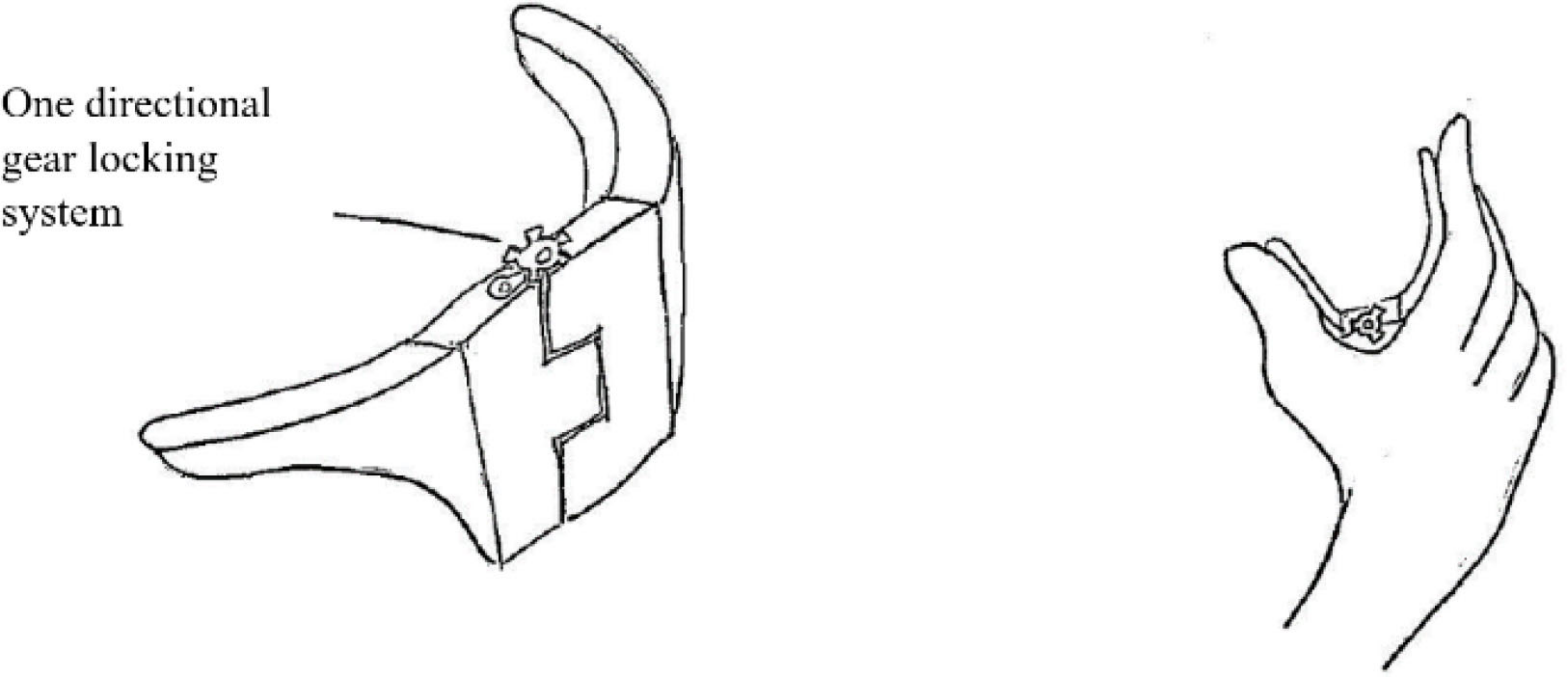
The other way around concept design.

Figure 10 shows a finger assistant powered by a miniature linear actuator. Based on the segmentation principle, weaker fingers could benefit from a device like this. The other way around principle suggested a concept that allows the elderly person to initiate the pinching action instead of relying totally on devices. Figure 11 shows a clamp device that has the ability to lock the pinched object in place after the pinch force is applied by the user. All contact points between the fingers and the device would have sufficient friction, as suggested by the sub-class 2.1.1. For sub-class 2.1.2, visual feedback substitution was also suggested and shown in Figure 7.

### 4.2. Concept screening

The TRIZ methodology was used up to this stage of the research. Five different concept designs have been generated using this problem-solving tool. The concept screening was based on a numerically weighted concept scoring method by Stuart Pugh. ^106, 107^ A set of general criterions ^108^ is directly adopted to avoid bias selection.

a. Criterion A - compatible with the overall task and with one another
b. Criterion B - fulfils demands of the requirements list
c. Criterion C - realisable in respect of performance, layout, etc.
d. Criterion D - expected to be within permissible costs
e. Criterion E - incorporates direct safety measures or introduces favourable ergonomic conditions
f. Criterion F - preferred by the designer’s company supervisor

Some modifications were made to the criteria to accommodate this project. Criterion A was defined in this study as the ability of a concept to be compatible with other concepts, or in other words, to be flexible. Criterion B was modified to represent the ability of a concept to solve the root of the problem. Criterion C was amended to reflect general feasibility, while criterion D was amended to reflect costing. Criterion E was modified to reflect safety measures. Criterion F was changed to reflect the preference of author PKN as an advisor, since a company does not exist in this study’s context. Table 3 presents the concept-screening matrix of the finger pinch enhancer for elderly people.

**Table 3.** The concept-screening matrix.

Concepts					
Selection criteria	Mechanical substitution & taking out	Dynamisation	Partial action	Segmentation (reference)	The other--way around
Compatibility/flexibility	0	+	-	0	-
Fulfils demands of requirements list	-	0	-	0	-
Realisable in principle	0	-	+	0	-
Within permissible cost	-	0	+	0	0
Incorporates direct safety measures	+	-	0	0	+
Preferred by PKN	-	-	-	0	-
Sum +'s	1	1	2	0	1
Sum 0's	2	2	1	6	1
Sum -'s	3	3	3	0	4
Net Score	-2	-2	-1	0	-3
Rank	3	3	2	1	4

*Notes: “+” is for better than; “0” is for same as; “-” is for worse than; PKN is the author.*

The design concept for segmentation was selected to be the reference for concept selection since there have been studies on finger rehabilitation devices that have used similar working principles, such as linkages and actuators. ^109– 112^ From the concept-screening matrix, it was found that the segmentation concept ranked the highest. This concept was brought forward to the product development stage as the main concept.

### 4.3. Product development

The segmentation concept suggested that in order to improve pinching abilities, weak fingers should be identified and strengthened. However, this concept still requires a product design method to advance from a conceptual idea into a feasible product.

Design methods are often seen as procedures or a set of tools used to assist design engineers. ^113^ These methods can be complex and vary for each design expert. Pugh ^106^ suggested a four-stage design process beginning with 1) exploration, 2) generation, 3) evaluation and 4) communication. Pahl, Beitz ^108^ also used a four-stage process but with a different classification; 1) conceptualising, 2) embodying, 3) detailing and 4) computing. Karl and Steven ^114^ introduced a similar but more customer-centric design process that allows each step to move flexibly rather than in a single direction as decisions often need to be revisited due to the complexity of the process.

The design mission statement, which was created to provide the general direction for the pinch enhancer, is shown in Table 4. The concept generation process in this research used a four-step method inspired by Karl and Steven: ^114^

1. Functional decomposition
2. Patent search
3. Systematic exploration
4. Concept selection

**Table 4.** Mission statement for finger pinch enhancer.

Mission statement: finger pinch enhancer	
Product description	• A portable device that assists elderly people during pinching actions
**Benefit proposition**	• Able to pinch more effectively with device
**Assumptions**	• Power-assisted • Lightweight • Device attached to the hands
**Target market**	• Senior citizens • People with weak finger pinching abilities

*4.3.1. Functional decomposition*

**Figure 12.**
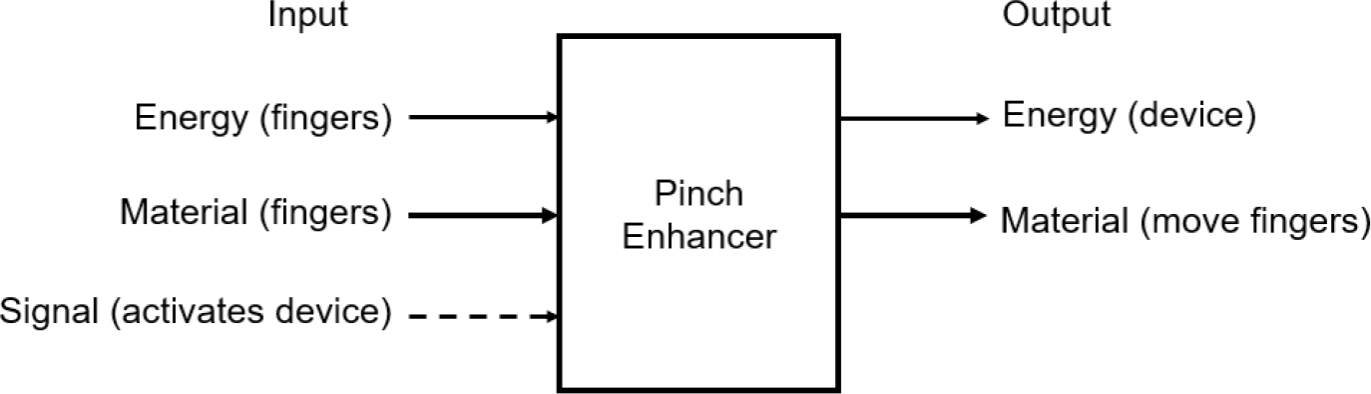
Functional diagram of a pinch enhancer.

Functional decomposition is a generic term used to describe the process of resolving a relationship into constituent parts such that the original function can be recreated from those parts. ^115^ It was used in this study to divide the problem into subproblems. The functional decomposition of the proposed finger pinch enhancer concept is shown in Figure 12. This functional diagram represents the initial problem described as a function of its energy, material, and signal flows.

**Figure 13.**
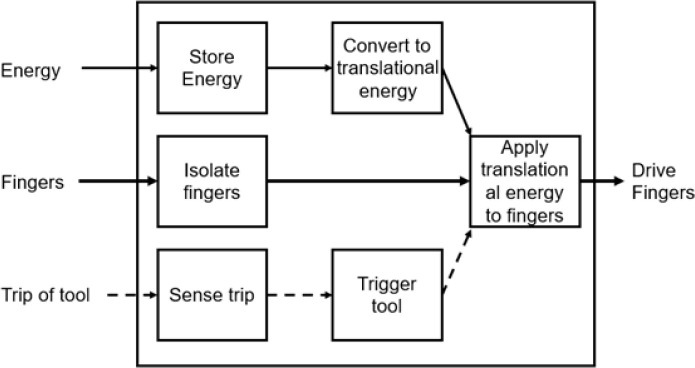
Functional decomposition diagram with subfunctions.

The functional decomposition was then divided into its subfunctions based on operations such as storing energy, isolating fingers and sensing a trip ( Figure 13). The smaller subfunctions represent elements that are needed for the product to function holistically. The signal, or trip of tool, describes components used to trigger the device to carry out programmed instructions.

*4.3.2. Patent search*

With the problem broken down into smaller sections, an external search was required to find existing solutions or possible concepts that address the overall problem or any of the subfunctions. This information-collecting process was executed through a patent search conducted on 29th December 2020. The inclusion criteria for the patent search comprised finger support, finger grip, finger pinch assistant, and pinch stabiliser, while the exclusion criteria comprised of finger exercise devices, arm exoskeleton, and stationary finger support. Google Patents was the search engine used for keyword searches. The keywords used were finger support, finger grip, and pinch stabiliser. Table 5 shows the function and strengths of the patents reviewed.

**Table 5.** Patents related to the pinch enhancer or any of its subfunctions.

No.	Name (citation)	Function	Strengths
1	Wedge gripper ^116^	Flexible finger held device which protects the fingers while angling or wedging a bar towards the palm	Effective with every cylindrical handle. Provides proper placement in the palm eliminating free rotation of a cylindrical bar.
2	Adaptive grip ^117^	Aiding users with limited hand use and dexterity to grasp everyday objects, has a finger grip, an anchor, and a clasping mechanism.	Can be used comfortably for everyday activities.
3	Portable hand rehabilitation device ^118^	A therapeutic device for improving voluntary control of paretic muscles in a patient’s extremity.	Portable device that can be strapped onto a patient's wrist. Uses somatosensory input as a functional guidance to improve motor function.
4	Actuated glove orthosis and related methods ^119^	An actuated glove orthosis used to strengthen a person’s hand.	Each finger digit having at least one mechanical stop. Hands free to grasp objects.
5	Hand exoskeleton force feedback system ^120^	A force feedback device for hand exoskeletons.	Force feedback unit and direct drive motor system controlled by a microcontroller.

*4.3.3. Systematic exploration*

Upon gathering many different conceptual solutions to the subproblems, three of the subproblems were chosen to be explored further, namely storing energy, sensing trip, and applying translational energy. These subproblems were chosen as they are the pillars of the product and greatly define the remaining subfunctions. As an illustration, if an external mechanical switch was chosen as the sense trip, then the trip tool could be a push start button while the method of storing energy could involve using dry cells or a suitably rated power supply. Table 6 shows a list of solutions for the three selected subproblems.

**Table 6.** Solutions for subproblems on storing energy, sensing trip, and applying translational energy.

Solutions to subproblem of storing energy	Solutions to subproblem of sensing trip	Solutions to subproblem of applying translational energy
Dry cells	Finger movements	Lever
Power banks	Push start button	Linkages
Wall socket power	Voice command	Gears
Pneumatic actuator		Slider
Chemical energy		Clasping mechanism
Solar power		Rollers
Human power		Wire cables

With the three listed subproblems and their respective solutions, there were a total of 147 possible combinations (7 × 3 × 7). It was almost impossible to explore all these options due to the amount of time and energy required. Therefore, a concept classification tree (see section 4.3.3.1) and concept combination table (see section 4.3.3.2) were used to streamline the process into a more focused and manageable direction.

*4.3.3.1. Concept classification tree*

**Figure 14.**
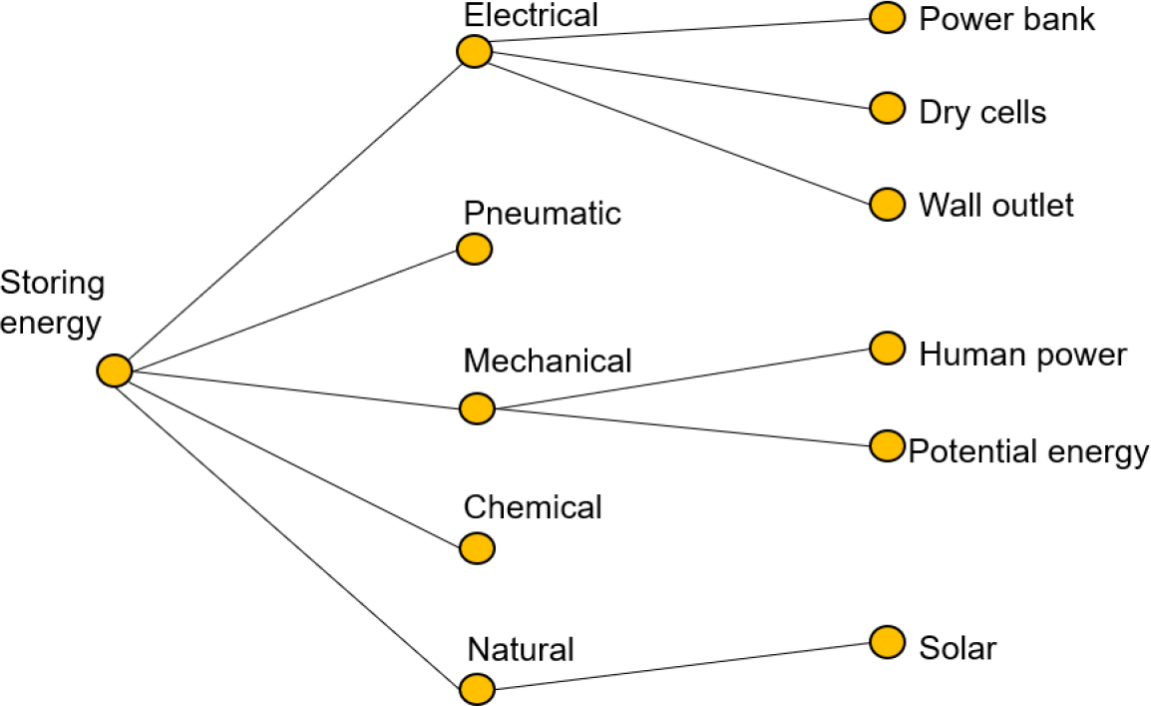
Classification tree for subfunction of storing energy.

The classification tree is often used in data mining to group cases or objects into classes of dependent variables. ^121^ The classification tree was used in this product development process to group solutions into classes for improved decision making. The selection criteria depended on the likelihood of the classification in constraining the remaining subproblems. Figure 14 shows the classification tree for storing energy.

**Figure 15.**
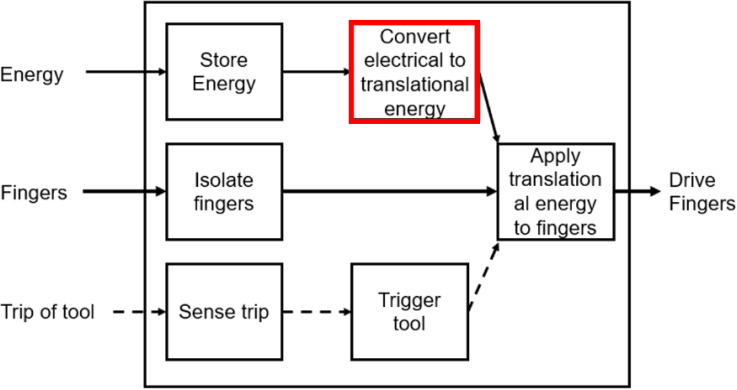
Refined functional decomposition diagram.

The pneumatic, chemical, and natural branches were disregarded from the tree, for being less portable, more expensive, and inefficient, respectively. In terms or refining the functional decomposition, the electrical method of storing energy was deemed the most suitable method. This decision was largely due to the fact that in order to store enough mechanical energy to move the fingers, a complex and bulky mechanism would be required. This limitation would violate the design needs of being lightweight and non-movement-restricting. Therefore, the functional decomposition diagram was refined as per Figure 15.

*4.3.3.2. Concept combination table*

Based on the refined functional decomposition diagram and the pruning steps from the concept classification tree, the concept combination table was used to match different solutions together to generate different concepts. Table 7 presents the three possible concept combinations of the three key subproblems.

**Table 7.** Concept combination table for pinch enhancer.

Combination	Convert electrical to translational energy	Sense trip of device	Applying translational energy
1	Linear actuator	Voice command	Linkage
			Linear transfer
2	DC motor	Push start button	Wire cables
3	DC motor	Finger movements	Linkage

**Figure 16.**
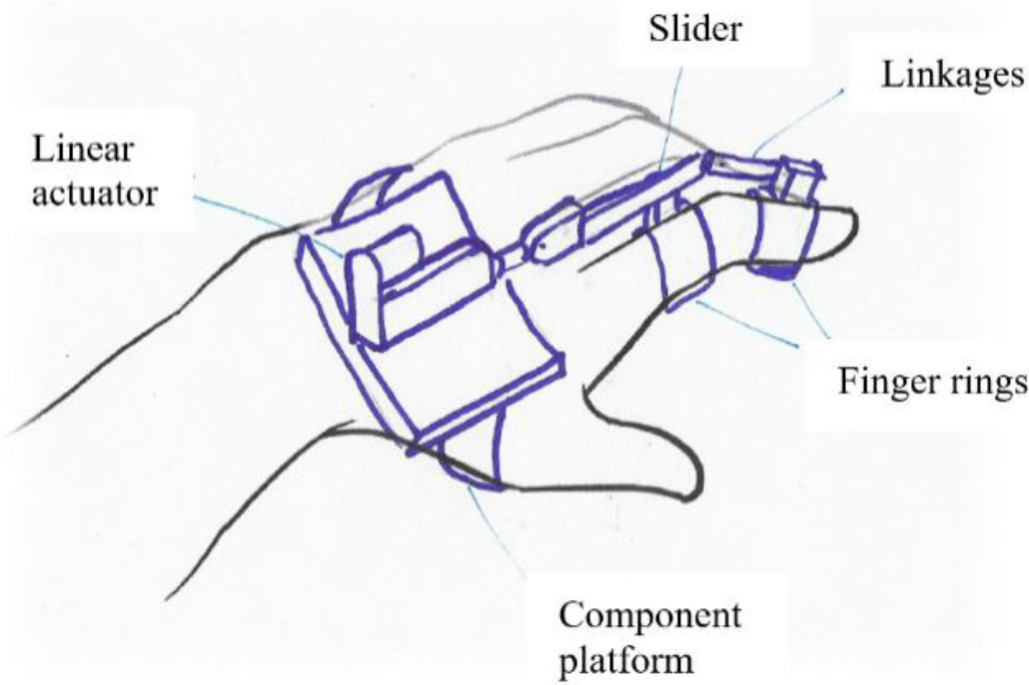
Concept 1 – Linkages to control finger movements.

**Figure 17.**
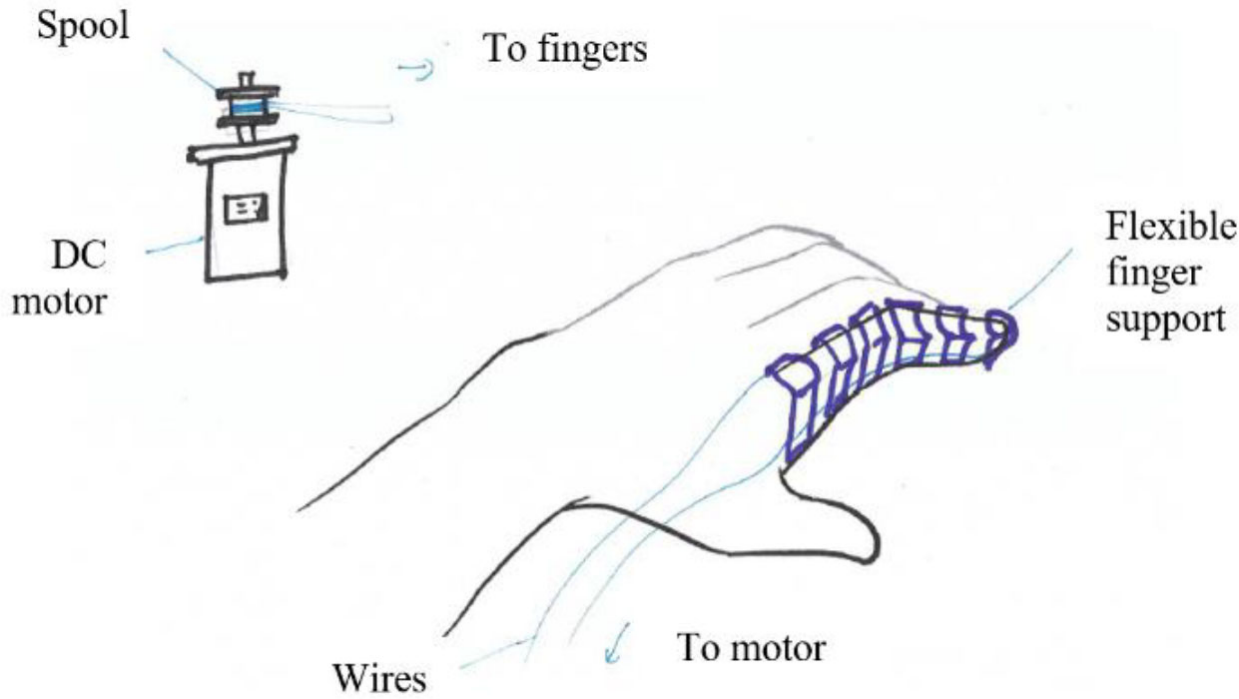
Concept 2 – Wires for flexible finger control.

**Figure 18.**
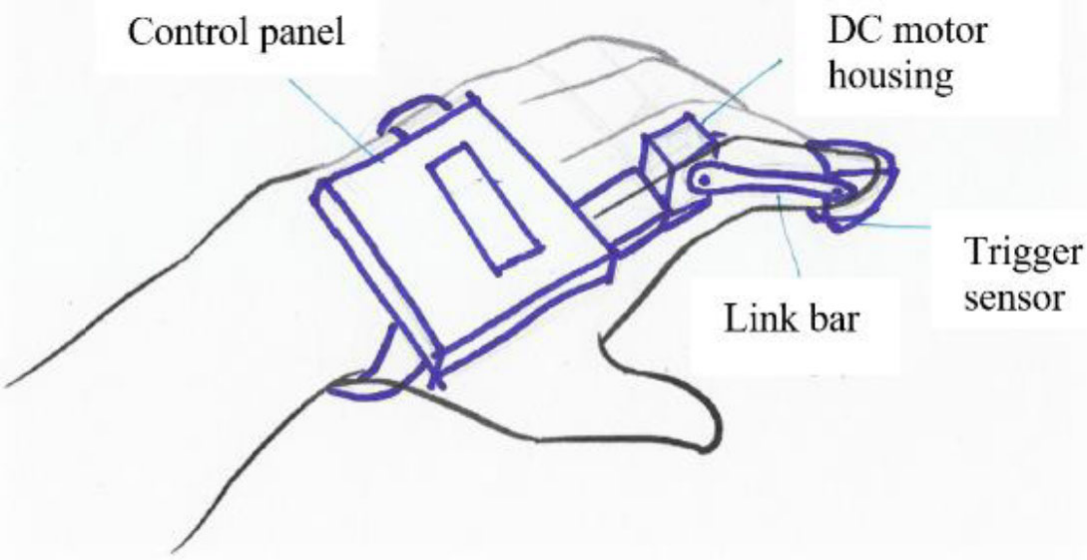
Concept 3 – Finger movements to trigger device.

The first combination uses voice command and linkages powered by a linear actuator (see Figure 16). The second combination uses a DC motor to actuate the fingers through tendon cables triggered by a push start button (see Figure 17). This type of exoskeleton design has the advantage of flexibility and safety. The third concept recommends a DC motor to actuate linkages to move the fingers for better finger control (see Figure 18). The activation of this device would be achieved through the sensing of finger movements (pressure sensors).

*4.3.4. Concept selection*

Concept selection is a process that refines and selects from the pool of generated concepts. A concept selection process was applied to the three generated concepts described in Table 7.

*4.3.4.1. Digital logic method*

A modified digital logic approach introduced by Dehghan-Manshadi, Mahmudi ^122^ was used to justify a reasonable selection. The digital logic approach has been used to rank material properties on a binary scale, where a score of one (1) is given to the more important property while zero (0) is given to the less important property. ^123^ The level of importance for each property is determined by the researcher or designer based on his/her self-assessment of the criterion. The modified approach ranks the more important property with a score of three (3) and the less important property with a score of one (1). However, when two properties have equal importance, they will both be given an equal numerical score of two (2). This method presents the advantage of keeping the least important property on the list.

In this study, the modified digital logic method was utilised with some adjustments. When two properties were compared in this study, it was challenging to consider them as equally important. Therefore, the numerical score of two (2) was not used the selection process. This condition, however, caused a large numerical gap to exist between the most important and least important properties due to the distance between scores one (1) and three (3). In the end, the score of two (2) was assigned to the more important property while the score of one (1) was given to the less important one. Table 8 shows the digital logic method in a pairwise table used to determine the weightage of each selection criterion.

**Table 8.** Pairwise comparison table of concept scoring selection criteria.

Criteria ( *n* = 6)	Number of Decisions [ *n* ( *n* – 1) /2]															Positive decisions	Weighing factors, *α*
	1	2	3	4	5	6	7	8	9	10	11	12	13	14	15		
Flexibility	1	1	1	1	2											6	0.133
Fulfils requirements	2					2	2	2	2							10	0.222
Realisable in principle		2				1				1	1	2				7	0.156
Cost			2				1			2			1	2		8	0.178
Safety				2				1			2		2		2	9	0.200
Preferred by PKN					1				1			1		1	1	5	0.111
	Total Number of Positive Decisions, *N*															45	1.00

*Notes: n – total number of criteria; 1 – Less important; 2 – More important; α = Positive decisions/N; PKN is the author.*

*4.3.4.2. Concept scoring*

With regard to concept scoring, the VDI Guideline 2225 published in 1964, ^124^ is the first catalogue of relative costs of materials. Although the guideline was meant to measure the costs of products in different size ranges, materials, and manufacturing process relative to a basic cost, it could also be used to rank value. Pahl, Beitz ^108^ used the VDI Guideline 2225 with a range of zero to four to evaluate different parameters along the design process. Table 9 shows the selection points and description used in the current study, which was also based on the VDI Guideline 2225.

**Table 9.** Point system based on VDI Guideline 2225. ^108^

Points	Meaning
0	Unsatisfactory
1	Just tolerable
2	Adequate
3	Good
4	Ideal

Table 10 presents the concept scoring matrix of the finger pinch enhancer for elderly people. The ratings were solely proposed by the main author based on his specific experience and knowledge on various hand and finger assistive devices. In reference to the main author’s superior design sense in the specific area of hand and finger assistive devices, the co-authors of this study concurred to the ratings and rankings provided by the main author. In this case, instead of selecting one reference concept, the reference for each criterion was selected individually as not one single concept was suited to be the reference. Finally, concept 2, which recommended the use of wires for flexible finger control, was selected based on its strengths in user comfort and ease-of-use.

**Table 10.** The concept scoring matrix.

Selection criteria	Weight	Concepts					
		1		2		3	
		Rating	Weighted score	Rating	Weighted score	Rating	Weighted score
Flexibility	0.133	**3**	0.399	4	0.532	2	0.266
Fulfils requirements	0.222	2	0.444	5	1.11	**3**	0.666
Realisable in principle	0.156	2	0.312	4	0.624	**3**	0.468
Cost	0.178	**3**	0.534	2	0.356	4	0.712
Safety	0.200	2	0.40	4	0.80	**3**	0.60
Preferred by PKN	0.111	2	0.222	**3**	0.333	4	0.444
Total score		2.311		3.755		3.156	
Rank		3		1		2	
Continue?		No		Develop		No	

## 5. Conclusion

Using the TRIZ approach, this study aimed to conceptualise finger grip enhancer designs that potentially facilitate elderly people’s day-to-day pinching activities. Using the segmentation principle, a finger assistant concept powered by a miniature linear actuator was selected. The concept recommended the use of a DC motor to actuate the fingers through tendon cables triggered by a push start button.

Using the TRIZ tool to identify root causes of degrading finger pinch function and generate solutions helped to unearth a more mechanical and product-oriented perspective of the biological challenge of finger weakness among elderly people. Although changes in the body would require physiological investigations, the systematic nature of TRIZ could lead researchers to solutions from other branches such as biomechanical, design and human factors engineering that may have been overlooked by experts of the field.

Poor finger pinch is an effect of ageing which negatively impacts elderly people’s quality of life. An assistive pinching device to aid in activities of daily living may be an effective option for them. Such a solution may give rise to implications related not only to the physical but also to the emotional and mental well-being of the elderly people in society.

### 5.1. Limitations and direction for future research

A potential limitation of this study was found in the product development stage. With the end goal of developing an assistive device for the fingers, it would have been advantageous to consider the inclusion of kinesiology studies on finger movements. The embodiment of kinesiology literature during the conceptualisation stage may have altered the dynamics of the ideation process for finger grip enhancers. However, this consideration was beyond the scope of this research project as the budget and resources available for this research did not account for finger movement test kits such as the Purdue pegboard, finger dexterity test kit or tweezer dexterity test kit.

Using the TRIZ recommended solutions and shortlisted concepts, this study could be extended to the product design and proof-of-concept stages. It would be viable and of interest for researchers to prototype and test this concept, which uses DC motors to actuate wires for the flexible control of fingers.

## Data availability

### Underlying data

Figshare: A TRIZ-driven conceptualisation of finger grip enhancer designs for the elderly. http://doi.org/10.6084/m9.figshare.14498649. ^125^

This project contains the following underlying data:

- Data Availability Sheet.pdf (descriptions of the literature and data sources used in CEC analysis).

Data are available under the terms of the Creative Commons Attribution 4.0 International license (CC-BY 4.0).
