# Supplementary material for: A TRIZ-driven conceptualisation of finger grip enhancer designs for the elderly
Source: F1000Res. 2021 May 17;10:392. [Version 1] doi: 10.12688/f1000research.51705.1 (PMC8290208; doi:10.12688/f1000research.51705.1)
Supplement: Peer review history

# Peer review: Reviewer response for version 1

**DOI:** 10.5256/f1000research.54897.r87151

**Published:** 2021-07-19

**Authors:** Shamsul Bahri Md Tamrin

**Affiliations:** 1 Department of Environmental & Occupational Health, Faculty of Medicine and Health Sciences, Universiti Putra Malaysia, Serdang, Malaysia

**Abstract: **

- The conclusion should be focusing on the finding and suggestions of the case study

**Introduction:**

1. Can you include pictures of several different types of pinching that can be performed?
2. **In the problem statement**, you did mention several studies on problem-solving related to pinching issues among the elderly, therefore can you mentioned the outcome and issues on some of the studies conducted and their solution previously?

**Literature review:**

1. Please double-check regarding carpal tunnel since carpal tunnel has a different etiology. Your study focused more on age-related than work-related.
2.  Subchapter on Factors contributing is not focused on what are the factors that lead to reduced pinch ability but more on the effect of age and reduction of systemic motor reduction. I advise revising with literature on factors or change the subtitle for this chapter.
3. Subchapter 2.4 – do you have any pictures of some basic exoskeleton.

**Methodology:**

1. 3.1.1 CEC Analysis – can you suggest the reference in developing CEC as suggested in Figure 3.
2. Figure 3 – does the color-coding have a different interpretation. Although described on the next page, in the figure legend, the figure should stand and be clearly understood.
3. In Figure 3, how do you verify the CEC? Each variable is cited through references or based on the opinion of the subject matter expert?
4. Table 1 – you had listed the source of examples in numbers. I suggest listing the name and numbers in easing the reading for readers.
5. In 3.1.2 – the 40 generalized recommendation is a default recommendation, or you had pre-selected or based on the 40,000 patents?
6. In an earlier study by (77)..I suggest changing, in the earlier study by Tan (77), so that the sentence is having much clarity.
7.  The root cause of contradiction 2 as you had mentioned is similar to the previous study, please mentioned which one that the root cause is similar to? And does it have the same inventive principles and system parameters?
8. Table 2 – I really don’t understand the mechanism and the method from the principle of engineering contradiction leading to how to do you select system parameters and inventive principle.
9. Table 2 – why the certain principle is bold? Is it automatically selected by TRIZ or manually selected by the author?
10. 3.1.2.1 – physical contradiction, the table below should be Table 4 under physical contradiction for each root cause?
11. 3.1.2.1 – physical contradiction, how do you categorize for list of segmentation, the other way around, and asymmetry?
12. Figure 10 – suggested sub-class 2.1.1 and 2.1.2 – what part of this sub-class? I did not find which one.

**Product development:**

1. There are several methods in design development, - which one from the study that you had emulate?
2. For Table 3 – maybe you should explain the calculation of the total rank score. How the score was made and what is the significant calculation of the score. Patent search – how do you come out with the right keywords for the patent search?
3. Concept scoring – in Table 8, the number of decisions – I need to understand where you have 10 observations. Please explain whether the decision was done by a single evaluator repeatedly or 10 different evaluators?
4. Table 10 – can you show an illustration of each concept? If not maybe only focus on concept 2 which had been selected.

**Conclusion:**

1. From the conclusion, do you mean concept 2?
2. How effective is the use of TRIZ method? And overall this study is a combination with TRIZ and the overall process of engineering in product development and not solely on TRIZ. 

Is the work clearly and accurately presented and does it cite the current literature?

Yes

If applicable, is the statistical analysis and its interpretation appropriate?

Not applicable

Are all the source data underlying the results available to ensure full reproducibility?

Partly

Is the study design appropriate and is the work technically sound?

Yes

Are the conclusions drawn adequately supported by the results?

Partly

Are sufficient details of methods and analysis provided to allow replication by others?

Yes

Reviewer Expertise:

Physical ergonomics, Industrial Hygiene and occupational health

I confirm that I have read this submission and believe that I have an appropriate level of expertise to confirm that it is of an acceptable scientific standard.

# Peer review: Reviewer response for version 1

**DOI:** 10.5256/f1000research.54897.r85521

**Published:** 2021-06-04

**Authors:** Leonid Chechurin

**Affiliations:** 1 Lappeenranta-Lahti University of Technology, Lappeenranta, Finland

The topic of the paper is important. Society is ageing and the more we help the elderly to be active members of the community and, more importantly, self-supportive, the more resources we have. I would also notice that the problem of finger grip enhancement is very important for children/people with special needs suffering from multiple sclerosis or CRH paralysis.

 The paper presents a story of enhancer design from the analysis of situation to the selection of the best to the criteria design. TRIZ, Root cause analysis, literature analysis and landscaping, functional analysis and decomposition and concept scoring matrix are used as design tools. 

 The paper has clear value from engineering point of view as it brings a story, develops a design case study and presents several concepts of finger grip enhancer. They can be used (and I have to say I already recommended the paper to several designers) by practicing engineers, physiotherapists and Do-It-Yourself enthusiasts who want to improve the life of people around them. 

 The paper has questionable scientific value, as it does not provide proofs that: 

1. These designs are actually helping, solving the problem or they are better in any way that any existing enhancers. 
2. The results are reproducible, the method of design is reliable.

In other words, it is very good that that paper presents an invention, a way of doing things, but the paper does not prove that this way of doing things is better than others. Would other design technics result in better designs? Would other engineers (not the authors) come to the same design if they use the same tools? These questions sound academic but that is why research papers are different to other texts.

 Maybe it would be good idea to change the title of the paper telling that it reports a Design Case Study, it reduces the scientific expectations of the reader.

**Other comments: **

- Abstract and page 3. Authors state that there is no study yet that applies TRIZ methodology to pinch function degradation. However, there are two publications by the same group of authors on this topic dated 2019 and 2021. Please, indicate what is different in the presented paper.
- Page 5. When a theory or a method is first introduced in a paper it is better to cite the founder of the theory. So, I believe it is fair to cite the first paper of Altshuller and Shapiro here, not a modern textbook (it can be recommended to the readers if the authors believe it is worth to).
- Page 7. When authors first time introduce Genrich Altshuller it is better to cite the original work, not a modern study with TRIZ application in mechanics.
- “suitable for new product innovation”. I would argue that TRIZ is the tool for invention, not for innovation. Innovation is different process of different scale.
- The authors used RCA for the analysis of initial situation, that is legitimate tool of cause. In modern TRIZ it is recommend providing the analysis of the situation with RCA (or better CECA) and with Function modelling. Function model makes a model of what is happening, a snapshot. While CECA often reveals cause + effect chains, that means we dive into the history.
- Figure 1 will be more informative if it is presented in input-output way. With current design and content, it doesn't give a visual explanation of the workflow.
- Page 5-6. I would anxiously say that what the authors use in their analysis is NOT cause-effect-chain-analysis of TRIZ, it is Root Cause Analysis, that was introduced by General Electric and by Lean/Taguchi (fishbone diagram). But, they all do the same job. The difference is that CECA uses operators OR and AND in the chains, that is really insightful sometimes. And CECA uses the term “Key disadvantage”, not “Root disadvantage” and have a reason for calling them so. I would recommend to the authors to go through original papers on CECA of Val Souchkov (Example Souchkov *et al*. (2005 ^1^)) or recent papers by Min Gyu Lee (S.Korea) (Example Lee *et al*. (2018 ^2^)).
- Figure 2. It is not exactly how contradictions are treated in classical TRIZ. Altshuller recommends resolving engineering contradiction with Contradiction matrix and those 40 inventing principles. But - he says - if we analyze the engineering contradictions deeper, we end up with physical contradictions, that are sharper (as they require THE SAME component of parameter to have opposite properties). Having formulated physical contradictions, we apply SEPARATION PRINCIPLES (one of 11 in classical TRIZ), not again 40 inventive principles. It is recommended to either reformulate the applied principles as separation (in time, in space, …) or at least give a comment on it.
- Page 16. It is interesting that the authors use Function Decomposition as product development tool. I would anxiously suggest that this approach, when a system is considered as a transformation unit for various flows (of energy, information, materials…) has the roots in NIST standards for functional basis in engineering design. Plz see and if you agree cite this document https://www.nist.gov/publications/functional-basis-engineering-design-reconciling-and-evolving-previous-efforts.
- It has already mentioned above (as well as by another reviewer) that the choice of the best concept seems to be subjective, it is based on author’s experience and preferences. It would have been much more reliable to collect as many as possible evaluations of the design(s) by other engineers, for example in a form of a survey.
- Authors reference medical literature that tend to update fast. However, over 50% of references are more than 5 years old.

Is the work clearly and accurately presented and does it cite the current literature?

Yes

If applicable, is the statistical analysis and its interpretation appropriate?

Not applicable

Are all the source data underlying the results available to ensure full reproducibility?

Partly

Is the study design appropriate and is the work technically sound?

Yes

Are the conclusions drawn adequately supported by the results?

Yes

Are sufficient details of methods and analysis provided to allow replication by others?

Partly

Reviewer Expertise:

Control and Oscillation theory, System modelling, optimization, Heuristic methods in design.

I confirm that I have read this submission and believe that I have an appropriate level of expertise to confirm that it is of an acceptable scientific standard, however I have significant reservations, as outlined above.

## References

[1] : Root Conflict Analysis (RCA ): Structured Problems and Contradictions Mapping. *Citesserx* .2005; Reference source

[2] : Introduction to cause-effect chain analysis plus with an application in solving manufacturing problems. *The International Journal of Advanced Manufacturing Technology* .2018;99(9-12) : 10.1007/s00170-018-2217-1 2159-2169 10.1007/s00170-018-2217-1

# Peer review: Reviewer response for version 1

**DOI:** 10.5256/f1000research.54897.r86104

**Published:** 2021-06-02

**Authors:** Adi Saptari

**Affiliations:** 1 Faculty of Engineering, Industrial Engineering Department, President University, Cikarang, Indonesia

This article has fulfilled certain criteria that make it a good research article, such as purpose, rigor, objectivity and replicability. The authors have shown comprehensive steps when applying the TRIZ method to come out with a reliable, conceptual design of a grip enhancer. One particular comment about what needs to be improved is on the screening of 5 different concepts. A set of general criteria were used, and modified. However, it was not explained in detail how the selection process was done, such as who are the evaluators and on what capacity. Furthermore, criteria F mentioned in the text was not clear, who is PKN? This step is key and significant for the next process of selection of the right concept chosen.

Is the work clearly and accurately presented and does it cite the current literature?

Yes

If applicable, is the statistical analysis and its interpretation appropriate?

Not applicable

Are all the source data underlying the results available to ensure full reproducibility?

Yes

Is the study design appropriate and is the work technically sound?

Yes

Are the conclusions drawn adequately supported by the results?

Yes

Are sufficient details of methods and analysis provided to allow replication by others?

Partly

Reviewer Expertise:

Optimization of linear and Non Linear Operations Research and human factors engineering

I confirm that I have read this submission and believe that I have an appropriate level of expertise to confirm that it is of an acceptable scientific standard.

# Response

**Published:** 2021-06-02

**Authors:** Poh Kiat Ng

**Affiliations:** Multimedia University, Malaysia

Many thanks to the reviewer for his constructive comments.

 Comment 1: “It was not explained in detail how the selection process was done, such as who are the evaluators and on what capacity”.

- Response to Comment 1: The ratings were solely proposed by the main author based on his specific experience and knowledge of various hand and finger assistive devices. This reasoning has been clarified in the last paragraph just before the conclusion.

Comment 2: “Who is PKN?”.

- Response to Comment 2: PKN is actually the second author of the article and the main supervisor of the main author. He is mentioned in section 4.2 as “the author PKN as an advisor”, which is reflective of the original criterion F “designer’s company supervisor”.

As such, no changes were made to the current article. But the authors appreciate the reviewer comments very much. Thank you.
